# Environmental Subconcussive Injury, Axonal Injury, and Chronic Traumatic Encephalopathy

**DOI:** 10.3389/fneur.2018.00166

**Published:** 2018-03-27

**Authors:** Wendy A. Morley

**Affiliations:** Thionetics, Toronto, ON, Canada

**Keywords:** subconcussion, axonal injury, chronic traumatic encephalopathy, lipopolysaccharide, environmental neurotoxins

## Abstract

Brain injury occurs in two phases: the initial injury itself and a secondary cascade of precise immune-based neurochemical events. The secondary phase is typically functional in nature and characterized by delayed axonal injury with more axonal disconnections occurring than in the initial phase. Axonal injury occurs across the spectrum of disease severity, with subconcussive injury, especially when repetitive, now considered capable of producing significant neurological damage consistent with axonal injury seen in clinically evident concussion, despite no observable symptoms. This review is the first to introduce the concept of environmental subconcussive injury (ESCI) and sets out how secondary brain damage from ESCI once past the juncture of microglial activation appears to follow the same neuron-damaging pathway as secondary brain damage from conventional brain injury. The immune response associated with ESCI is strikingly similar to that mounted after conventional concussion. Specifically, microglial activation is followed closely by glutamate and calcium flux, excitotoxicity, reactive oxygen species and reactive nitrogen species (RNS) generation, lipid peroxidation, and mitochondrial dysfunction and energy crisis. ESCI damage also occurs in two phases, with the primary damage coming from microbiome injury (due to microbiome-altering events) and secondary damage (axonal injury) from progressive secondary neurochemical events. The concept of ESCI and the underlying mechanisms have profound implications for the understanding of chronic traumatic encephalopathy (CTE) etiology because it has previously been suggested that repetitive axonal injury may be the primary CTE pathogenesis in susceptible individuals and it is best correlated with lifetime brain trauma load. Taken together, it appears that susceptibility to brain injury and downstream neurodegenerative diseases, such as CTE, can be conceptualized as a continuum of brain resilience. At one end is optimal resilience, capable of launching effective responses to injury with spontaneous recovery, and at the other end is diminished resilience with a compromised ability to respond and/or heal appropriately. Modulating factors such as one’s total cumulative and synergistic brain trauma load, bioavailability of key nutrients needed for proper functioning of restorative metabolic pathways (specifically those involved in the deactivation and clearance of metabolic by-products of brain injury) are key to ultimately determining one’s brain resilience.

## Introduction

Chronic traumatic encephalopathy (CTE) is conceptually a devastating neurodegenerative disease that develops later in life in susceptible individuals with history of repetitive head injury. Early in 2011, researchers at the VA-BU-CLF Brain Bank, the largest tissue repository in the world focused on traumatic brain injury (TBI) and CTE (1), concluded that CTE “is a neurodegenerative disease that occurs later in the lives of some individuals with a history of repeated head trauma. The exact relationship between repetitive mild traumatic brain injury, with or without symptomatic concussion, and CTE is not entirely clear, although it is possible that repetitive axonal injury sets up a series of metabolic, ionic and cytoskeletal disturbances that trigger a pathological cascade leading to CTE in susceptible individuals” (2). There has been intense medical and scientific focus on this issue and the field of CTE research in general, and more specifically, efforts to better understand the risk of repetitive brain trauma and its relationship to CTE causation. Recent reports have been critical of initial studies noting flaws in the reporting of CTE cases (inconsistent definition) and the scientific process (selection bias of autopsy cases and absence of healthy controls), thereby prompting much controversy and debate within the medical and scientific communities (3, 4). Most of the controversy stems from the use of samples of convenience rather than random, which inherently limits the ability to accurately determine the prevalence of CTE in the general or specified population. However, that being what it may, the original studies and their conclusions seem to be consistent with mounting evidence that supports a role for repetitive axonal injury from concussions, with and without clinically evident symptoms, and the consequent downstream neuroimmune activation, ionic shifts, and cellular flux has on toxic proteinopathies such as amyloid beta (Aβ), tau and TAR DNA-binding protein 43 (TDP-43), cognitive dysfunction, and impaired neurotransmission that are features classically seen in CTE (2). TDP-43 is an important RNA-binding protein, which appears to partly colonize with p-tau, accumulating in the frontal and temporal lobes. This mislocation and accumulation of TDP-43 is a classic feature of CTE, with >85% of cases presenting with some level of abnormal accumulation, and severe deposits present in advanced cases. The location of this TDP-43 pathology has a deleterious effect on personality, behavior, and cognitive function (5, 6). Two diverse patterns related to Aβ emerge in chronic cases of CTE; approximately 50% of cases present with significant diffuse amyloid plaque, while the remaining cases are essentially devoid of diffuse amyloid plaque (2, 7). CTE also presents with significantly more white matter injury than seen in a number of neurodegenerative diseases (3). This may be a function of the prominent axonal injury associated with the secondary biochemical cascades seen in conventional concussion from biomechanical force (contact sports, military activities, and motor vehicle accidents) (3, 8, 9). Subconcussions, defined as “blows to the head generating enough force to disrupt neuronal integrity without resulting in clinically evident symptoms” (3, 10), are a relatively recent focus of research. With the advent of helmet technology capable of measuring the magnitude of force, studies are now providing evidence that athletes who are exposed to multiple hits of <15 g-force over a season experience similar brain damage (affecting neuronal health and white matter integrity, and impairing cognitive function) as those with concussion (3, 10, 11). While some may argue it is not clear if the g-forces measured from the helmets correlate to the g-forces applied within the brain, this line of evidence underscores the notion that subconcussions are much more damaging than once thought (10, 11), and one could argue they are in fact a subcategory of brain injury. Based on this new level of insight on brain damage from subconcussions, scientists are now taking a broader view of CTE etiology by stating that “CTE appears to be most correlated with ‘total lifetime brain trauma,’ which can be defined as a combination of subconcussive brain trauma and concussions” (12), and this is best measured as a cumulative dose of exposure over a lifetime (11).

It is important to note that, while there has been considerable progress made on understanding the correlation between repetitive brain injury and CTE, the context of this understanding is limited to repetitive brain trauma from biomechanical forces such as motor vehicle accidents, contact sports, and military activities. Of particular relevance to understanding CTE susceptibility, specifically among those who develop CTE apparently in the absence of any overt repetitive brain trauma is an independent stream of research that shows similar brain damage coming from non-biomechanical forces, such as environmental toxins and endotoxins (9, 13). Brain insults arising from these external factors appear to follow the same pathophysiology of glutamate excitotoxicity, tauopathy, mitochondrial, and oxidative dysfunction that is seen in secondary injury from conventional brain injury (9, 13, 14). Furthermore, environmental toxins and bacterial endotoxins seem to have profound synergistic properties that magnify their effects even at seemingly inconsequential exposure levels (7). If brain trauma from both biomechanical and non-biomechanical forces exerts its effect *via* neuroimmune activation mechanisms of secondary brain injury, then it is plausible that any event generating enough effect, either additive or synergistic, to trigger neuroimmune activation, whether biomechanical in nature or not, should be accounted for when considering an individual’s history of repetitive brain trauma. The physiological interplay involved in CTE is complex, and many critical questions remain. Cases with history of (clinically evident) repetitive brain injury and yet void of any CTE pathology are also of interest, and speak to the needed elucidation of the mechanistic roles of neurodamage versus neuroprotection in CTE susceptibility.

This review article explores two novel concepts: environmental subconcussive injury (ESCI), a term coined by the author to depict environmental toxicant exposure sufficient to stimulate neuroimmune activation, ionic shifts, and other secondary neurochemical events resulting in axonal injury, and diminished brain resilience (DBR), a concept first presented by the author in 2014 to describe a specific physiological state of nutritional functional deficiencies and altered microbiome created by modern-day exposures and lifestyle choices that lead to a diminished capacity to respond and recover from brain insults (15).

In this review article, the author explores the question “*do individuals with high level of exposure to environmental toxins have different brains or physiology that in turn affect how they respond to further brain insults and injury?*”

Before going into the discussion of how ESCI is involved in brain resilience, it is worthwhile to review the current understanding of the physiological response to brain injury. Damage from brain injury occurs in two phases. The initial phase is the occurrence of injury itself and is where axonal shearing and tissue damage is often recognized as a direct consequence of the initial force of impact, especially from a rotational force (8). The second phase of brain damage comes from a cascade of neurochemical events that begin at the time of injury and continue for days, weeks, or even months (11, 16). This ongoing secondary phase results in delayed axonal white matter injury with a greater number of axonal disconnections than is seen during the initial injury, and it is synonymous with secondary brain damage (8).

Axonal white matter injury, also referred to as traumatic axonal injury or diffuse axonal injury, is evidenced by impaired white matter structural integrity, axonal stretching, swelling, and ultimately disconnection. This occurs across the spectrum of brain trauma severity, including mild traumatic brain injury (mTBI) and subconcussion (3, 8). Axonal injury (secondary brain damage) is more often functional in nature, resulting from altered cellular or physiological milieus such as ionic shifts, altered metabolism, and dysfunctional neurotransmission. It strongly correlates with cognitive dysfunction, poorer quality of life, and an increased risk to subsequent brain injury, the latter perhaps due to slower reaction time among other factors (8, 17). Microstructural injury or secondary brain damage is generally not visible with mainstream computed tomography imaging and diagnostic magnetic resonance imaging but is detectible with more sensitive imaging technology such as diffusion tensor imaging (DTI), which is better at detecting microstructural changes of white matter following brain trauma. Unfortunately, DTI is currently used mostly in a research rather than clinical setting (11, 17).

As the secondary cascade of neurochemical events and injury progresses, clinically relevant symptoms associated with early stages of CTE emerge revealing difficulties of concentration, disorientation and dizziness, impaired memory/cognition, and affective disorders such as impulsivity, aggression, depression, suicidality, and ultimately diminished motor control (3).

## Concussive Biomechanical Force: Mechanisms of Injury

Brain injury activates an early and primary (innate) immune response dominated by two activation events of microglia, the brain’s dominant immune cells (18), and toll-like receptors (TLRs), specialized proteins critically involved in the innate immune system’s response to danger (19–21). The immune response can be described as a double-edged sword, with one side mounting a rapid and largely destructive response to injury or invading pathogens, and the other side providing a restorative, cooling down process that returns physiological homeostasis after the danger has passed. In the case of brain trauma, these early and primary activation events are critical for mTBI-induced inflammatory signaling for tissue repair, removal of debris, neurogenesis, and eventual dampening of the immune response. The immune system, however, also responds rapidly to danger from either injury or invading pathogens with the release of neurotoxic substances, extracellular and intracellular accumulation of toxic glutamate and Ca++, respectively, blood–brain barrier (BBB) breakdown and reactive oxygen species-induced lipid peroxidation (LP) (16, 21, 22). The complex interplay of multiple destructive post-injury response events sets the stage for profound energy crisis and impaired glucose metabolism, which occurs acutely 3–5 days post-injury (17).

## Neuroimmune Activation, Ionic Shifts, and Cellular Flux

As described earlier, the initial reaction to brain injury is an immune response. The inflammatory response to brain trauma is associated with activation of innate immunity, specifically *via* TLRs. TLRs are pattern-recognition receptors that respond to pathogens *via* pathogen-associated molecular patterns (PAMPs) and tissue injury *via* damage-associated molecular patterns (DAMPs). In response to central nervous system (CNS) infection and/or injury, PAMPs and DAMPs bind to TLRs to elicit an immune response (23). TLR4, the most studied TLR, has evolved as the receptor that responds to pathogenic infections, specifically *via* interaction with lipopolysaccharide (LPS), a potent endotoxin of Gram-negative bacteria (24). What is now increasingly coming to light is that TLR4 responds to BOTH pathogenic infection from Gram-negative bacteria/LPS AND tissue injury from TBI with strong evidence that TLR4 activation is pivotal in brain trauma and the secondary neuroinflammatory events that lead to CNS neurodegeneration and neural injury (21, 24). TLR4 activation is also implicated in astrocyte signaling, a step critical for tissue repair/scarring and neurogenesis post TBI (16, 21, 25). In the brain, microglia primarily express TLR4, the activation of which stimulates microglia (26) to start the deleterious neurochemical cascade of events post-injury. TLR4 is also expressed by astrocytes and neurons among brain cells (21). TLR4 activation and its pivotal role in response to brain trauma have profound implications, which will be made clear shortly, to increased risk of DBR, vulnerability to subsequent brain insults, and downstream neurodegeneration in pathologies such as CTE.

Another primary immune event is microglia activation. One of the most profound consequences of this early and primary event is increased glutamate levels from indiscriminate efflux of massive amounts of glutamate from both activated microglia and astrocytes, as well as impaired glutamate homeostasis and clearance from dysfunctional glutamate transport systems. Glutamate is the primary excitatory neurotransmitter in the CNS and is critical for normal brain functioning including memory, cognition, and learning. However, in high amounts, extracellular glutamate is toxic, leading to tissue injury *via* excitotoxicity (27). Accumulation of excessive extracellular glutamate over-stimulates glutamate receptors, increasing axonal membrane permeability and triggering a toxic increase of intracellular Ca++. This indiscriminate efflux of glutamate followed by an influx of Ca++ is a critical step in glutamate excitotoxicity, which is central to secondary axonal injury. This is a delayed degenerative process that involves, among other things, lethal metabolic changes, free radical generation, and mitochondrial dysfunction, which ultimately leads to axonal disconnection and neuronal death (8, 9, 15).

Magnesium, one of the brain’s most abundant ions, is essential in virtually every biochemical and physiological function including oxygen uptake, energy production, metabolite transport, and as an ionic gradient, to name a few (15, 28). Research shows that the increase in intracellular Ca++, and its subsequent accumulation in mitochondria, is essential for the efflux of magnesium from the mitochondria (29). Of clinical relevance, a rapid and prolonged drop in magnesium is correlated with a significantly worse prognosis (15), perhaps as a result of the generation of cytokines that stimulate α-amino-3-hydroxy-5-methyl-4-isoxazole propionic acid receptors, making cells more sensitive to excitatory neurotransmitters like glutamate (15, 30). Moreover, as described in Section “Energy Crisis and Reduced Glucose Metabolism,” magnesium is needed to stabilize ATP and create bioavailable energy.

Cholesterol loss is also known to follow glutamate-mediated excitotoxicity and requires high levels of intracellular Ca++ (31), a recognized state in the secondary neurochemical cascade of brain trauma (32). This “dumping” of cholesterol has significant implications to concussion pathophysiology, yet is rarely, if ever, mentioned. Cholesterol is a major regulator of neural function and synaptic transmission and a key component of lipid rafts, which among other things are involved in endocytosis and intracellular signaling for cell survival. Reduced cholesterol impairs glutamate intracellular storage in vesicles, and membrane cholesterol loss inhibits synaptic transmission (31).

## Energy Crisis and Reduced Glucose Metabolism

As with the pattern of ionic shifts noted above, there is a well-documented pattern of energy demand and glucose metabolism that follows brain injury (17, 33). Post-injury, there is an acute and intense increased demand for energy as ATP-dependent pumps rev up in an attempt to stabilize ionic gradients perturbed by the injury. The increased demand for energy from ATP-dependent pumps creates a state of hyper or intense cerebral glucose metabolism precisely when there is significantly reduced cerebral blood flow (CBF). This mismatch between supply and demand leads to a profound energy crisis (17).

Moreover, there are other contributing factors that increase the demand for ATP and/or reduce the synthesis or availability of ATP/cellular energy thereby exasperating the energy crisis. One such factor is cholesterol, which is critical for, among other things, proper neurotransmission and appropriate storage of glutamate within cellular vesicles. In the adult brain, neurons rely on astrocytes to facilitate synthesis of cholesterol, an energy intense process, which adds to an already high demand post trauma (31, 34), which is likely one of the reasons there is a proliferation of astrocytes observed post-injury. In 2012, Sodero et al. postulated that “at least some of the typical signs and symptoms of the pathological conditions that are accompanied by excessive glutamatergic neurotransmission such as stroke, epileptic seizures, or TBI, may be due to glutamate-triggered cholesterol dyshomeostasis” (31).

On the other end of the equation of supply and demand is the supply or bioavailability of enough ATP to meet the increased energy demand. A few factors come into play in this regard. Magnesium is a cofactor of ATP, making it critical for ATP bioavailability (35). Like cholesterol, studies show a rapid and prolonged decline of this important ion, post-injury. The increased demand of magnesium for ATP bioavailability occurs when it is in short supply, a mismatch of supply and demand like that of the energy crisis. This drop in magnesium supply would pragmatically lead to a situation where even if adequate amounts of ATP were being produced, the lack of magnesium would essentially add to the energy crisis, with insufficient levels of bioavailable ATP. Fluoride, a ubiquitous chemical is also known to inhibit ATP bioavailability (36).

The brain depends on a steady supply of glucose for proper metabolism, and CBF is critical for aerobic glucose metabolism (37). After acute injury, increased glycolysis with resulting pyruvate and lactate production is seen. This increased glucose metabolism is in line with the increased demand for energy; however, efficient ATP synthesis requires glucose and oxygen, both of which are in short supply post-injury (32). Therefore, in a state of compromised CBF, the normal aerobic process of ATP production is severely compromised. Brain injury uniquely challenges the normal cerebral metabolism creating a state known as “hyper-glycolysis,” a term used to describe the level of metabolic crisis occurring after acute injury (37). As a potential explanation of the pyruvate and lactate seen after concussion, an alternative mechanism that does not require oxygen seems possible. Generating only 6 ATPs as compared with 30 ATPs when metabolized aerobically in the mitochondria, anaerobic glucose metabolism within the cytoplasm is clearly much less efficient. Interestingly, with the help of amyloid beta, which is uniquely able to stimulate lactate dehydrogenase, pyruvate is broken down into lactate *via* anaerobic fermentation, which is capable of producing significantly more ATP (38). Because lactate is an energy form that is useable by neurons, it has been suggested that the increased lactate produced post trauma may be shuttled to neurons as a way to offset the lack of oxygen (32, 38). This alternative energy mechanism involving amyloid beta has some intriguing implications from a plausible defensive strategy deployed to address the post trauma energy crisis, and may in fact serve as a neuroprotective aspect of amyloid precursor protein (APP), discussed in more detail below, as a mechanism to lessen the energy crisis and thus reduce the level of cell death.

Finally, unlike other cells in the body, brain cells rely on glycolysis for energy because they are unable to directly convert or utilize fatty acids (fats) as an alternative energy source. While fatty acids do not cross the BBB, ketone bodies, which are water-soluble molecules synthesized in the liver from fatty acids, do. Therefore, ketone bodies represent another alternative fuel for the brain in its compromised state post-acute injury (39). Further, some interesting research from University of California, Los Angeles suggests that the brain is not static in its preferential use of fuel, but rather responds rapidly and dynamically to neuropathologic conditions such as TBI, by shifting to be more receptive to ketone metabolism (40).

Taken together, the state of energy crisis or hyper-glycolysis, and the level of efficient shift into alternative energy sources, has a profound impact on one’s ultimate risk and vulnerability to subsequent injury.

## ROS-Induced LP

Lipid peroxidation, a further consequence of glutamate efflux and Ca++ influx, is considered a “hallmark” and one of the most significant pathophysiological processes involved in TBI (22, 41). Significant amounts of free radicals, both ROS and reactive nitrogen species (RNS), are created *via* multiple mechanisms, and these are among the most noted aspects of secondary injury in TBI (22). Notably, RNS peroxynitrite contributes significantly to LP-induced damage in acute brain injury as well as ROS acrolein and 4-hydroxynonenal, one of most toxic aldehydic by-products of LP. These reactive species serve to further intensify ROS/RNS production, significantly worsening the situation (22, 42). LP-induced damage includes but is not limited to:

- Cellular and mitochondrial impaired function.
- Impaired glutamate transport mechanisms, worsening glutamate-mediated excitotoxicity.
- Impaired Ca++ homeostasis, as well as mobilization of Ca++ from intracellular stores.

Glutathione, the brain’s primary antioxidant, is part of a critical system involved in neutralization and clearance of reactive species and xenobiotics, respectively. The Nrf2 pathway regulates antioxidant processes, such as those in the glutathione and thioredoxin/peroxiredoxin systems *via* cooperative cross talk between neurons and astrocytes: neurons responding with activity-dependent antioxidant support and astrocytes with intrinsic antioxidant support (43). The relationship between post-injury increases in ROS and RNS production and the countering function of the Nrf2 pathway elucidate the significant increased demand for glutathione in brain trauma.

## Two Fates of APP

Amyloid precursor protein, which is most often associated with the damaging effects of Aβ seen in Alzheimer’s disease, is interestingly also acutely upregulated post-acute injury, and this acute upregulation is considered to be neuroprotective posttrauma. Upregulated APP is also considered to be a sensitive marker of axonal injury (33, 44). This cellular response is an important one as APP metabolism has two fates. The first is an amyloidogenic pathway, which leads to the production and accumulation of neurotoxic Aβ, and the other is a non-amyloidogenic pathway, which is highly neuroprotective against excitotoxicity. The neuroprotective mechanism is likely due to APP’s ability to activate potassium channels thereby reducing cellular Ca++ influx, protecting against hypoglycemic damage, and limiting induction of ROS, among other things. The APP pathways are dependent on enzymatic action, specifically soluble APPβ for amyloidogenic metabolism and soluble APPα for non-amyloidogenic metabolism. Docosahexaenoic acid (DHA) and heparan sulfate (HS) proteoglycans are critically important for APP’s neuroprotective pathway, and DHA supplementation is shown to increase the neuroprotective effect of sAPP, which is correlated to its ability to bind to heparin (44). Functional deficiencies in the above noted nutrients or genetic polymorphisms might explain the inconsistent pattern of amyloid beta plaque seen in CTE cases.

## ESCI: Mechanisms of Injury

Many studies show systemic brain inflammation is intimately connected to neurodegenerative disease. While the mechanisms of the response have yet to be elucidated, studies are now showing an innate immune response to chemical exposure to be associated with increased extracellular glutamate, oxidative stress, and LP, which are events consistent with the cascade of neurochemical events responsible for secondary brain injury (9, 13).

Here, the author outlines a novel concept that the central mechanism of secondary brain damage from environmental forces is *via* an indirect route of altered microbiome and increased levels of the endotoxin, LPS. LPS is a virulent component of Gram-negative bacteria, which elicits a strong immune response. As mentioned earlier, TLRs are pattern-recognition receptors that respond to pathogens *via* PAMPs and tissue injury *via* DAMPs. In response to CNS infection and/or injury, PAMPs and DAMPs bind to TLRs to elicit an immune response (23). TLR4, the most studied, has evolved as the receptor that responds to pathogenic infection, specifically LPS from Gram-negative bacteria (24).

There is mounting evidence that chemicals, especially environmental toxins, have a deleterious effect on the human microbiome, by shifting the balance of gut bacteria toward higher levels of Gram-negative bacteria (45–48). Higher populations of Gram-negative bacteria naturally increase the amount of circulating LPS, which is believed to activate the innate immune response and cascade of neurochemical events. The author suggests that both streams of innate immune response (DAMP and PAMP) map to the juncture of microglia activation after which they follow the same progressive secondary neurochemical events, albeit from slightly different mechanisms of TLR4 activation (direct versus indirect). Restated, tissue injury from biomechanical force stimulates TLR4 *via* DAMP, while pathogen LPS (indirectly from environmental exposure) stimulates TLR4 *via* PAMP. After microglial activation, the mechanisms of secondary brain injury are consistent between both types of brain injury.

With respect to neurological TLR4 activation from LPS, it is important to note that a fundamental debate has centered on whether LPS and other TLR4 ligands cross the BBB and directly activate TLR4s or activation is *via* some other mechanism (24). In 2012, studies by a Swedish scientist, Carina Mallard, suggested that “TLR4 ligands do not seem to cross the BBB” but rather “cells of the BBB synthesize immune signal molecules to activate cells in the CNS in response to peripheral LPS” (24). In support, evidence shows that endothelial cells in the BBB seem to be the main target of neuroimmune activation, and the presence of TLR4s on these endothelial cells is required for chronic brain inflammation from LPS exposure (24, 49).

Most studies looking at the relationship between LPS exposure and brain inflammation from glutamate and calcium flux, study the response when LPS is delivered directly to CNS tissue. What is of particular relevance to this discussion are multiple studies that document an opening up of both the BBB and gastrointestinal (GI) barrier within hours post-acute injury. GI permeability is thought to be triggered by vagus nerve stimulation while BBB permeability is thought to be enhanced as a mechanism to allow systemic immune mediators to cross the BBB into the CNS (50–53). In the presence of an altered microbiome, an acute injury from biomechanical force would likely have profound implications with respect to LPS translocating from the gut directly into the brain. Regardless of the pathway of TLR4 activation from LPS, there are numerous studies that support this connection between LPS and neurological inflammation. Borges et al. showed microglia activation in the brain with chronic exposure to LPS (54), and others showed a rapid rise in intracellular Ca++ in microglia, a primary event during brain injury, after systemic exposure to LPS (55).

In short, environmental toxins are now shown to alter the human microbiome largely through their preferential antibiotic effect on Gram-positive bacteria, such that the microbiome balance swings to be Gram-negative dominant. This imbalance increases the load of LPS the body is exposed to. Peripheral LPS either stimulates the BBB immune signaling molecules to activate TLR4 *via* PAMP or is translocated in the presence of a compromised (open) GI barrier, and thus initiates the neuroimmune response within the CNS. The response to ESCI is strikingly similar to that mounted after tissue injury from biomechanical force. Specifically, microglial activation is followed closely by glutamate and calcium flux, excitotoxicity, ROS/RNS generation, LP, and mitochondrial dysfunction and energy crisis, among other events.

**Figure 1:**
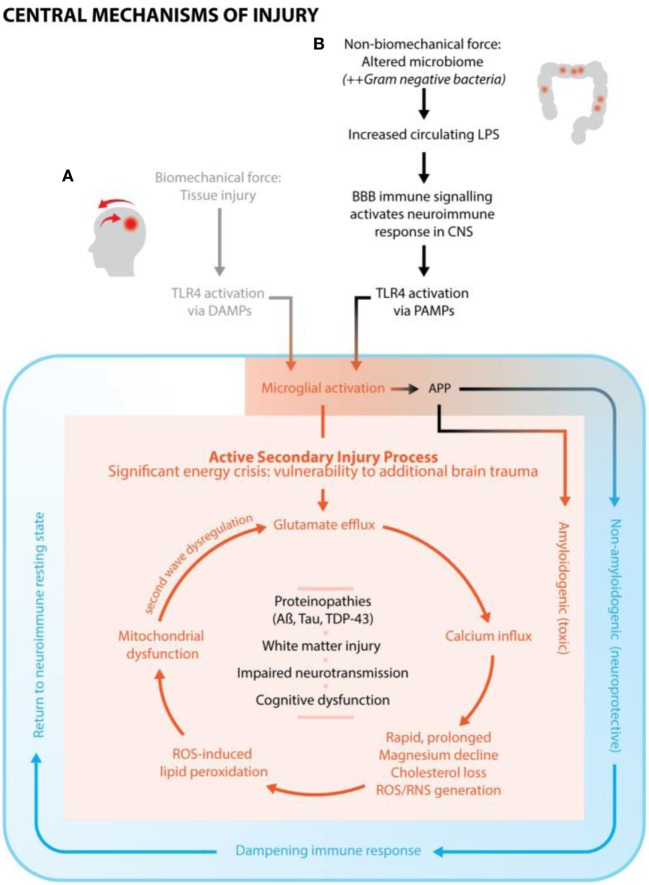
A conceptualized framework of brain injury, cross-linking subfields of neuroscience, nutrition, and environmental toxicology. **(A)** Biomechanical force causes tissue damage (primary injury) and initiates neuroimmune response. Secondary injury, with a characteristic cascade of neurochemical events, occurs from the point of primary injury and continues for days, weeks, or months. Over the course of injury, the neuroimmune response goes from fully activated, to an immune dampening state, to finally a return to resting state. **(B)** Altered microbiome/increased circulating lipopolysaccharide (LPS) is hypothesized to be the primary injury, which activates the neuroimmune response, including the classic secondary injury features and neurochemical events as seen with conventional brain trauma. It is conjectured that this type of injury is most associated with chronic, subclinical neuroimmune activation.

In summary, Figure 1 illustrates the mechanisms of injury from non-biomechanical force (ESCI) demonstrating how ESCI maps into the same pathway of secondary brain injury as from biomechanical force once past the juncture of microglial activation.

## Discussion

This review article synthesizes important scientific literature cross-linking the subfields of neuroscience, nutrition, biochemistry, and environmental toxicology. The result of this lateral approach is the revelation that brain trauma due to environmental toxins may follow a very similar mechanism of secondary injury as that of conventional brain trauma.

Like with conventional biomechanical brain trauma, ESCI damage occurs in two phases. The primary damage is the occurrence of injury to the microbiome *via* exposure to microbiome-altering toxins, and the secondary damage results from the cascade of neurochemical events that occur from the point of TLR4 activation, which can persist indefinitely. Below, four broad categories of microbiome-altering events that lead to primary damage are discussed: environmental toxins, traditional antibiotic therapy, additives, and lifestyle factors.

### ESCI—Primary Damage

#### Environmental Toxins

Two environmental toxicants, glyphosate and fluoride are discussed below. Glyphosate, the most widely used herbicide is primarily used for two functions (15, 56). Glyphosate’s primary function is as an herbicide. However, more troubling is that it is now being used for desiccation, a process of drying out grains before harvest (15, 57). When glyphosate is applied, it accumulates on plants and cannot be removed by washing or cooking, and it remains stable for a year or more (58). Recent studies are documenting increased concentrations of the herbicide in human food supplies, the meat of slaughtered animals, and in humans who follow a standard or conventional diet (57–59). In a recent study by an independent, Food and Drug Administration-registered laboratory, glyphosate residues measured 1,125.3 ppb in General Mill’s Cheerios, a popular breakfast cereal, and this figure is over 60% higher than the level of 700 ppb set in the USA as the maximum glyphosate permitted in tap water. This same study (59) reported that a popular product labeled as certified to be non-genetically modified (non-GMO) had levels as high as 812.53 ppb that also exceeded the maximum tap water threshold. More foods with glyphosate residue increase the risk of unintentional overexposure and consequent microbiome injury. The implications to health from the added use of glyphosate for non-agricultural purposes, such as desiccation, may be significant.

Fluoride is another ubiquitous toxicant in the environment (60). It is added to public water supplies, included in dental hygiene products as well as used in dental treatments with the goal to reduce tooth decay (61, 62). While most fluoride is excreted, some accumulates in the body. The regular use of products containing fluoride may therefore also increase the risk for unintentional overexposure. Fluoride accumulation is shown to calcify bones and interestingly the pineal gland (63, 64). The clinical relevance of calcified pineal gland has not been fully elucidated; however, it poses an interesting question given the pineal gland’s role in sleep regulation regarding a potential correlation because sleep disruption is a characteristic symptom associated with many neurodegenerative diseases, as well as acute brain trauma (65–67). More research seems indicated.

Of particular relevance to ESCI is how environmental toxins disrupt the human (and animal) microbiomes. In 2010, Monsanto was awarded a patent (first submitted in 2003) for glyphosate as an antibiotic. Since then, studies have confirmed its antibiotic effect, specially highlighting most beneficial bacteria (typically Gram-positive) to be more sensitive to glyphosate’s antibiotic effect, and conversely most pathogenic bacteria (mostly Gram-negative) to be more resistant to the effect. With a balance tipping to more pathogenic/Gram-negative bacteria and fewer beneficial bacteria, there are more opportunistic pathogens and increased LPS from the Gram-negative bacteria. Of particular interest is testing that showed that *Bifidobacterium adolescentis*, a beneficial strain of bacteria, which increases mucosal function and is particularly protective against intestinal LPS (68), is highly sensitive to glyphosate (45, 68). Moreover, antimicrobial effects of fluoride also indicate a strong effect against Gram-positive bacteria (69). Glyphosate and fluoride are two ubiquitous chemicals whose exposure risk is insidious in nature.

#### Traditional Antibiotic Therapy

Many beneficial Gram-positive bacteria within the human microbiome are particularly vulnerable to pharmaceutical antibiotics (70). That said, there is no debate that responsible use of antibiotics saves lives. However, the larger concern with respect to risk factors of ESCI is the common agricultural practice of using antibiotics prophylactically. A report published in 2015 cited global use of antibiotics in food animals to be estimated at 63,000 t in 2010 with an anticipated 27% increase of consumption by 2030. While there has been significant attention to the risk of this widespread practice of prophylactic antibiotic use on farms and antibiotic resistance, accurate measurement of usage remains challenging (71, 72).

#### Additives

There are two categories of additives worth mentioning: additives in food and additives in medicines.

Processed foods are laden with chemical additives used to improve taste, texture, stability, and shelf-life. But these culinary benefits come at a price. Emulsifiers are chemical additives that make food products more appealing by making ingredients blend together. Used often in ice cream, dressings, cream cheese, and baby formula, emulsifiers are ubiquitous in processed foods. In a study on chronic inflammatory diseases and intestinal microbiome, researchers noted consumption of popular additives such as polysorbate 80 and carboxymethylcellulose are known not only to impact mouse but also human microbiomes (47). Artificial sweeteners, another class of popular food additives, have been widely studied, and consumption of which is linked to altered microbiome (dysbiosis) (48). Furthermore, aspartame specifically seems to play an important role in excitotoxicity with the suggestion that the artificial sweetener may directly act on glutamate *N*-methyl-d-aspartate receptors (73).

The second important category of additives is medical additives. Vaccine adjuvants are used to generate an enhanced immune response to the specific antigen of the vaccine (74). Recently, TLR4 agonists administered *via* vaccines have been described as “souped-up” vaccines. Monophosphoryl lipid A, a chemically modified product of LPS, was the first TLR4 agonist approved for use in human vaccines, and it is currently included in Cervarix™, a vaccine for human papillomavirus (HPV) and FENDrix™, a vaccine for hepatitis B (75). While pharmaceutical organizations consider the use of TLR agonists as adjuvants to have improved “overall safety profiles” (75), the concern is that they are designed specifically to elicit a strong immune response *via* the same TLR4 mechanisms as is activated in brain injury. To be clear, the author is not suggesting vaccines cause ESCI, but vaccines with TLR4 adjuvants would reasonably be expected to exacerbate TLR4 activation, especially if exposure coincides with microbiome alterations (increased LPS).

#### Lifestyle Factors

Briefly, two common lifestyle factors are worth mentioning given their roles in altering the microbiome balance: high fat diets and alcohol use. Current popular opinion has latched onto the mantra that low-fat/high-carb diets are the culprit for rising obesity rates and promoting high fat, high protein, and low carb diets. While obesity is well known to be associated with chronic low-grade inflammation, the mechanism and origin of inflammation has been elusive (68). However, a study by Kim et al. investigated the role of a high fat diet on LPS induced inflammation *via* TLR4 activation. Their results showed that a high fat diet altered gut microbiome by preferentially increasing Gram-negative bacteria and plasma LPS (68). Furthermore, the study documented a significantly reduced population of bifidobacteria, the bacterial strain associated with increased mucosal barrier function and protection against LPS, as noted in relation to glyphosate exposure (45, 68). It is important to note that there is considerable research relating to the neuroprotective health benefits of a ketogenic diet on neurological health, which seems in contrary to the abovementioned study. The type of fat used in the study of Kim et al. was not detailed. It is quite conceivable that the fats used may have been industrial-grade fat sources, which are typically soy or canola based and known to be produced from GMO/glyphosate crops. If so, the altered microbiome may have been a function of the type of fat used, versus fat itself. Further studies using different fat sources are needed.

Alcohol consumption, albeit at physiologically relevant concentrations, has been shown to activate microglia *via* TLR4 pathways and contribute to neuroinflammatory damage (18). Studies have also documented alcohol’s effect on altering the microbiome, again *via* a pattern of preferentially destroying Gram-positive bacteria, thus enabling the overgrowth of Gram-negative bacteria. It has also been established that heavy alcohol use enhances translocation of LPS out of the gut and into the general circulation thereby increasing plasma concentrations, likely due to its action on bowel inflammation and disruption of the GI barrier (76, 77).

Taken together, there are considerable every-day environmental and lifestyle exposures that alter the microbiome balance and increase plasma LPS, which have implications of cumulative and synergistic load exposure.

### ESCI—Secondary (Brain) Damage

As described earlier, secondary brain damage from ESCI appears to follow the same neurodamaging pathway as secondary brain damage from conventional brain injury, once past the juncture of microglial activation. Here, we outline how the added variable of LPS within the neurochemical cascade of secondary events enhances volatility of response, specifically with respect to neurodegenerative disease.

Two fundamental concepts in ESCI are microglial activation or immune priming and the synergistic relationship between LPS and glutamate excitotoxicity. Recall that microglia are the primary immune cells of the brain, and their primary function is to modulate the inflammatory response to injury or pathogens. As shown in Figure 1, activated microglia and increased pro-inflammatory cytokines in response to excitotoxins can lead to neurodegeneration. Microglia are fundamentally in one of three states: inactive (ramified/resting), intermediate activation (primed), or fully activated (predominantly neurodestructive) (7). Multiple types of brain insults including environmental toxicants such as herbicides and fungicides, LPS, and brain trauma all shift microglia (7) from its natural resting position to a vigilantly primed state. In this altered state, the microglia respond to subsequent injury in a more rapid and virulent manner than when in their original resting state. The significant work of Blaylock on immunoexcitotoxicity (7) elucidates the synergistic properties of LPS and excitotoxicity, specifically documenting how compounds such as glutamate, aluminum, and LPS on their own are subtoxic, but when combined become completely neurotoxic as evidenced by the presence of excitotoxic lesions. Fluoride, aluminum, and combined fluoride/aluminum are also shown to increase ROS and the subsequent generation of lipid peroxide by-products (14) consistent with mechanisms of secondary brain injury. Furthermore, LPS involvement is being increasingly correlated with both clinical pathological findings and neurodegenerative diseases that present as comorbidities to CTE. There are documented correlations between LPS and amyotrophic lateral sclerosis, Lewy body disease, Parkinson’s disease, amyloid beta and TDP-43 to name a few (78–80).

## Modulating Factors: Neuroprotective or Neurodamaging

The human body has an exquisite array of protective and restorative mechanisms in place to maintain homeostasis and health. For instance, the human microbiome naturally includes both Gram-negative and Gram-positive bacteria and is equipped with the means to quickly recognize LPS (endotoxin) and to effectively deactivate and detoxify (eliminate) it. Critical control mechanisms involve various compounds, such as LPS-binding protein, which is needed to recognize LPS; serum lipoproteins (low-density lipoprotein and very low-density lipoprotein), which are needed to neutralize the toxin (81–84); and acyloxyacyl hydrolase enzymes, which also inactivate LPS (82, 85). Regarding LP, the enzyme glutathione-*S* transferase 4 conjugates and detoxifies the toxic by-products of oxidative stress and LP after acute injury (41, 86).

Many, if not all, homeostatic mechanisms involved in capacity to clear and detoxify LPS, as well as damaging by-products of TLR4 activation, excitotoxicity, free radical generation, and LP have multiple points of failure along their metabolic pathways that relate to nutrients. For instance, alkaline phosphatase is an enzyme that is also involved in neutralizing LPS (87) and is zinc dependent (88). Interestingly, low plasma alkaline phosphatase is associated with decreased activity of tissue non-specific alkaline phosphatase in the brain post-acute injury and is thought to be involved in the characteristic accumulation of tau proteins (tauopathy) seen in CTE (89). Magnesium is critical for ATP bioavailability, and the neuroprotective effect of APP is reliant on its binding to HS. Restated, a functional deficiency of a critical nutrient could cause a failure of a given metabolic pathway. That said, effective diagnostic testing of functional nutrient status is imperfect, and many nutrients have a stringent span between deficiency and toxicity. Table 1 offers a summary of how various homeostatic mechanisms map to relevant modulating factors involved in secondary brain injury.

**Table 1:** Modulating factors of recovery.

Biochemical event	Modulating factor	Modulating effect
LP	Glutathione-*S*-transferase 4	Deactivation and detoxification of the LP by-product 4-HNE
	MTHFR	MTHFR is the main metabolic pathway to produce glutathione, the brain’s primary antioxidant. GsTa conjugates to glutathione which reduces toxicity of 4-HNE

Excitotoxicity	MTHFR	Low glutathione creates a state of low antioxidant activity, which results in increased oxidative stress. Higher oxidative stress results in reverse transport of glutamate thereby generating higher extracellular levels
	AOAH	Deactivation/detoxification Gram-negative by-product (LPS)
	Zinc deficiency	Alkaline phosphatase, which detoxifies LPS (the toxic by-product which triggers TLR4 activation), is zinc dependent

Cholesterol dysregulation	ApoE	ApoE is the principal cholesterol carrier in brain
		Lipoproteins are involved in LPS neutralization
		Poor toxin clearance is associated with ApoE4

APP/Aβ	DHA deficiency	Increased secretion of sAPPα leads to increased neurotoxic Aβ
	HS deficiency	Binding of HS to APP is required for neuroprotective effects

Energy crisis	Mg deficiency	ATP must bind to Mg to make it bioavailable

*4-HNE, 4-hydroxynonenal; MTHFR, methylterahydrofolate reductase; GsTa, glutathione-S-transferase class alpha; AOAH, acyloxyacyl hydrolase; LPS, lipopolysaccharide; sAPPα, soluble APPα; TLR, toll-like receptor; APP, amyloid precursor protein; DHA, docosahexaenoic acid; LP, lipid peroxidation; HS, heparan sulfate; Aβ, amyloid beta*.

## Conclusion

As noted above, this review article incorporates insights across multiple scientific bodies of knowledge to form a unique perspective on the epidemiology of CTE especially relating to the cumulative dose of exposure to brain trauma over a lifetime. However, it is important to note that while lateral thinking and cross pollination of concepts is compelling from a discovery perspective; the resulting conclusions are initially very much theoretical in nature and represent a call for appropriate research rather than a finding by itself.

In response to the question “*do individuals with high level of exposure to environmental toxins have different brains or physiology, which in turn affects how they respond to further brain insults and injury*,” the author has detailed pivotal mechanisms of altered physiology brought on by environmental toxicants that would result in a hyperresponse to subsequent brain injury, in individuals with high cumulative or synergistic levels of environmental toxicant exposure and/or significant modulating factors related to brain resilience.

**Figure 2:**
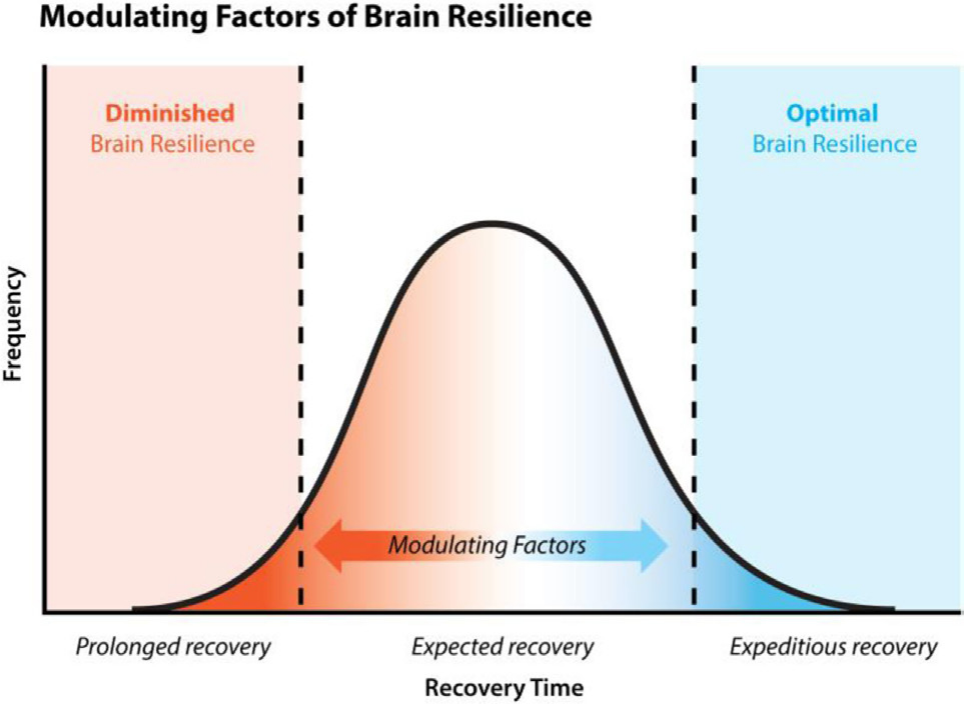
Brain resilience and recovery time. Normal clinical recovery of concussion is expected within 7–14 days, post-injury. 10–15% of concussed individuals experience persistent symptoms, known as post-concussion syndrome, lasting >3 months. Statistics are lacking regarding the percentage of concussed individuals who experience extremely fast resolution of symptoms 1–7 days. Brain resilience is a concept that considers various physiological states such as nutritional deficiency and the individual’s ability to detoxify toxic by-products like lipopolysaccharide, etc., and their impact on (a) the functioning of various mechanisms of recovery, (b) the speed of recovery, and (c) the efficacy of recovery. These various physiological states impact one’s brain resilience and therefore act as modulating factors to recovery.

Diminished brain resilience, a concept lightly touched on in this paper, describes a constellation of factors that conceivably predisposes one to increased susceptibility to various brain injury and downstream neurodegeneration (Figure 2). Susceptibility to brain trauma and downstream neurodegenerative diseases, such as CTE, can be viewed on a continuum of brain resilience. On one extreme is optimal brain resilience, characterized by normal state of low neuroimmune activation or resting microglia, with efficient responses to brain injury and/or insult. Optimal brain resilience would be reasonably expected in individuals who consciously take steps to reduce the cumulative and synergistic load of both concussive and subconcussive head blows and to ESCI by proactively managing genetic polymorphisms (e.g., methylenetetrahydrofolate reductase/B vitamin status), maintaining a properly balanced microbiome by limiting microbiome-altering agents, and by following an organic, non-GMO, well-balanced diet high in quality fruits and vegetables, thus ensuring optimal nutrient bioavailability and efficient functioning of innate homeostatic mechanisms.

On the other extreme would be a state of chronic primed microglia, characterized by hyperreactivity to brain injury and/or insult and a diminished capacity to recover; this state could be called DBR. Presumably, a person with unidentified (and therefore unmanaged) genetic polymorphisms, high exposure to microbiome-altering events with a lack of appropriate interventions to maintain microbiome balance, multiple nutrient functional deficiencies, and adherence to a standard American diet that is high in processed non-organic, GMO industrialized foods, and/or mechanical head trauma would be at a significantly greater risk of a DBR.

In conclusion, ESCI is a new concept that may have important implications for secondary brain injury from environmental factors, which could materially impact an individual’s CTE risk as ESCI increases their total lifetime brain trauma load along with subconcussive and/or concussive injury.

## Future Studies

This ESCI review article and the author’s previous work on DBR offer a novel hypothesis that could elucidate why those with seemingly no history of brain trauma develop CTE, and others with a history of repetitive brain trauma do not. It is the hope that the ideas captured within this review spark useful dialog within the research community leading to the undertaking of empirical research needed to determine the veracity of the hypothesis. Studies looking at the specific mechanisms of how environmental factors might lead to long-term sequelae similar to TBI sequelae are needed. While empirical research is the goal, this domain is complex and fraught with challenges. Three overarching limitations are problematic. First, the level of reliability and validity of nutritional and toxicant assessments required for empirical research may be inadequate. The human body is known to sequester various micronutrients and toxins as a defense mechanism when in a state of excess or deficiency. It is unclear if levels found within plasma, urine, or hair are an accurate measurement of toxic load or nutritional status. Levels may be skewed higher in the case of increased excretion or lower in the case of sequestering. Second, research into subconcussive injuries is still fledgling with important questions remaining. Specifically, it is unknown if the body’s response to chronic subconcussive insults follows the exact same immune-based neurochemical events as those involved in acute TBI and brain trauma. Many homeostatic mechanisms have biphasic responses to stimuli; one level creates an adaptive response, and a different level creates a protective response. How does this concept of biphasic response figure into subconcussive injury versus acute TBI. Finally, the sheer number of modulating factors acting on a large number of variables would require significant funding to facilitate the required complex research designs and analysis methods.

## Author Contributions

WM has reviewed the scientific literature, wrote the manuscript, and has read and approved the final version of the manuscript.

## Conflict of Interest Statement

The author declares that the research was conducted in the absence of any commercial or financial relationships that could be construed as a potential conflict of interest.
